# An Enzyme-Induced Novel Biosensor for the Sensitive Electrochemical Determination of Isoniazid

**DOI:** 10.3390/bios7020021

**Published:** 2017-06-05

**Authors:** Rajasekhar Chokkareddy, Natesh Kumar Bhajanthri, Gan G. Redhi

**Affiliations:** Electroanalytical Laboratory, Department of Chemistry, Durban University of Technology, Durban 4000, South Africa; chokkareddys@gmail.com

**Keywords:** enzyme-based biosensor, isoniazid, cyclic voltammetry, differential pulse voltammetry, pharmaceutical samples

## Abstract

In this present work, a glassy carbon electrode (GCE) was modified primarily with multiwalled carbon nanotubes (MWCNTs) and a composite of MWCNTs and titanium oxide nanoparticles (TiO_2_NPs). The enzyme horseradish peroxidase (HRP) was immobilized to enhance the sensing ability of GCE. The proposed biosensor was used for the sensitive determination of isoniazid (INZ) in various pharmaceutical samples. The electrochemical behaviour of the developed MWCNT-TiO_2_NPs-HRP-GCE biosensor was studied by using cyclic voltammetry (CV) and differential pulse voltammetric (DPV) techniques. Fourier transform infrared spectroscopy (FT-IR), X-ray diffraction (XRD), thermogravimetry (TGA) and transmission electron microscopy (TEM) techniques were used to characterize the developed sensor. Phosphate buffer solution (PBS) with pH 7 was used as supporting electrolyte in the present investigation. The cyclic voltammetric results revealed that the increment of anodic peak currents for the enzyme-induced sensor was almost 8-fold greater than that of a bare GCE. The DPV technique exhibited good limit of detection and limit of quantification values, viz., 0.0335 μM and 0.1118 μM, respectively. Moreover, the developed sensor showed long-lasting stability and repeatability without any interferents. This strongly indicates that the fabricated sensor shows outstanding electrochemical performance towards INZ, with excellent selectivity and sensitivity. The developed sensor was successfully applied to pharmaceutical samples and gave good percentages of recoveries.

## 1. Introduction

Pyridine-4-carboxylic acid hydrazide (INZ) is commonly used in chemotherapy for pulmonary tuberculosis along with pyrazinamide, rifampicin and ethambutol [1]. However, regular overdoses of INZ cause hepatotoxicity and may lead to death [2]. The World Health Organization (WHO) suggested that a daily dose of INZ per adult should be 4–6 mg/kg of their body weight [3]. INZ possesses both bacteriostatic and bactericidal action to interfere with the metabolism of nucleic acids, bacterial proteins, carbohydrates, and lipids. Hence, INZ plays a vital role in the therapeutic treatment of tuberculosis disease. Therefore, there is scope to develop a simple, rapid and robust method for the sensitive determination of INZ in various pharmaceutical samples. As per previous literature reports, INZ was analysed by using high performance liquid chromatography [4], capillary electrophoresis [5], fluorimetry [6], titrimetry [7,8], chemiluminescence [9] and electrochemical methods [10]. Electrochemical methods showed superior advantages like rapid, low-cost instrumentation, less time-consuming analyses and simple sample preparation, with reduced chemical consumption [11].

In electrochemical methods, the modification electrode results in the enhanced of sensitivity of the electrode. Various types of electrode coating agents like gold and platinum core shell nanoparticles [12], thionine-immobilized multi-walled carbon nanotubes [13], mercury film silver [14], poly3,4-ethylenedioxy thiophene films [15], modified multiwalled carbon nanotubes [16], and graphene oxide poly arginine [17] have been used for the determination of INZ. However, the above mentioned methods showed a lack of fast electron-transferring ability. Hence, there is a need to search for new nanocomposites, which exhibit good sensitivity with fast electron-transferring ability for the determination of INZ. Multiwalled carbon nanotubes (MWCNTs) exhibit larger surface area and high electronic conductivity by nature, resulting in good conductivity. MWCNTs are used in various batteries, supercapacitor electronics, and sensors [18]. TiO_2_NPs possess more advantageous characteristics like low cost, nontoxicity, long-term stability, and multi-functionality. Therefore, they are widely used as antiseptics, in anti-bacterial compositions, and as catalysts to promote various electrochemical reactions [19]. MWCNTs are combined with TiO_2_NPs and formed as an innovative class of carbon-based functional nanomaterials with good electrical, optical and catalyst properties [20]. The enzyme horseradish peroxidase (HRP) shows greater affinity for coupling with the nanocomposite for electrochemical transduction due to the presence of amino groups in the HRP enzyme. In addition, HRP has good catalysing properties, as well as a strong oxygenation nature for oxidization of a wide variety of organic substrates [21]. Moreover, HRP immobilized with a nanocomposite results in the formation of a biosensor with high stability and good efficiency.

The present work is designed and based on the fabrication of GCE with new generation nano-structured composites like the MWCNT-TiO_2_NPs-HRP composite for the sensitive determination of INZ. Furthermore, the modified sensor was applied to pharmaceutical samples to evaluate the realistic performance of the proposed sensor.

## 2. Experimental

### 2.1. Apparatus

The 797 VA Computrace (Metrohm, Herisau, Switzerland) with 1.3.1 software was used for the electrochemical investigations of INZ. A conventional three-electrode system with a glassy carbon electrode (GCE) or modified electrode was used as a working electrode, whilst a Ag/AgCl (3 M KCl) reference electrode and platinum wire were used as an auxiliary electrode. A Crison micro pH 2000 digital meter was used to prepare the supporting electrolyte. A Varian 800 Scimitar Fourier transform infrared spectroscopy (FT-IR) (SMM Instruments. Durban, South Africa) was used for the FT-IR characterization. A Transmission electron microscopy (TEM) JEM 2100 with a Lab 6 emitter (Max oxford instrument, JEOL Inc., Peabody, MA, USA) was employed for the study of surface morphology of the nanoparticles and biosensor. A Labcon 5019U ultra sonicator (Lasec, Durban, South Africa) was used for the ultrasonification of reagents. A Thermo Gravimetric Analysis (TGA) Differential Scanning Calorimetry (DSC) 1SF model 1346 (Columbus, OH, USA) with a STAR^e^ software version 9.20 (Mettler Toledo) instrument was used for the thermogravimetric characterization of the nanocomposite and the biosensor.

### 2.2. Chemicals and Reagents

All the regents used in this experiment were analytical reagent grade. Isoniazid and MWCNTs (Outer diameter (O.D).-L 6–9 nm–5 μM, catalogue number 724,769) were purchased from Sigma Aldrich (Durban, South Africa). The MWCNTs were prepared using chemical vapour deposition with cobalt, and molybdenum as a catalyst. Sodium dihydrogen orthophosphate, disodium hydrogen phosphate, sodium hydroxide, sulphuric acid, titanium tetra chloride, urea and N, N-dimethyl formamide (DMF) were purchased from Capital lab suppliers (Durban, South Africa). Alumina powder (3.0 μM) was purchased from Metrohm. The stock solution of 1 M INZ was prepared by dissolving the appropriate amount in a 50-mL volumetric flask. Upon serial dilution, working standard solution was also prepared. All the working standard solutions were prepared with deionized water, and kept in a refrigerator at 4 °C to ensure stability. According to the previous literature [22] with slight modification, 25 mL of TiCl_4_ was made up to a 100 mL volumetric flask with deionized water, in an ice cold bath. Likewise, 13 g of urea was dissolved in another 250-mL volumetric flask with deionized water. The urea solution was then slowly added to the contents of TiCl_4_, and then subjected to heating at 110 °C on a hot plate, with a magnetic stirrer for about 30 min. After the completion of the reaction, a white colloidal solution was obtained. The colloidal solution was centrifuged at 1000 revolutions per minute with rotational centrifugal force for 15 min. After centrifugation of the product, the residue was washed repeatedly with deionized water, and then dried at 60 °C for 3–4 h. The newly formed TiO_2_NPs were then stored in the refrigerator at 4 °C, for further use. A stock solution of INZ (1 M) was prepared by dissolving an appropriate amount in a 50-mL volumetric flask. Upon serial dilution, working standard solutions were made. Phosphate buffer solution (PBS) of 0.1 M with pH 7.0 was used as a supporting electrolyte throughout the experiment.

### 2.3. Fabrication of Nanocomposite Electrode

Prior to modification, the bare GCE was polished to a mirror-like surface with alumina slurry on a wet polished cloth and then further washed with deionized water. A total of 5 mg of MWCNTs was dissolved in 5 mL of DMF and thereafter kept for ultrasonification for about 30 min at 50 °C. The suspension was used for the modification of the GCE. Further, 10 mg of MWCNTs and 10 mg of TiO_2_NPs were dissolved in 10 mL of N,N-dimethyl formamide (DMF) followed by ultrasonification for about 1 h, to form a stable black suspension of MWCNTs-TiO_2_NPs. This was then used for the modification of the GCE. The MWCNT modified GCE and MWCNT-TiO_2_NPs modified GCE was prepared by dropping 5 µL of MWCNT suspension onto the cleaned electrode, which was then dried at 50 °C for 10 min.

### 2.4. Preparation of the MWCNT-TiO_2_NPs-HRP-GCE Enzyme Electrode

The HRP enzyme was immobilized on the MWCNT-TiO_2_NPs nanocomposite fabricated on the surface of GCE. The MWCNT-TiO_2_NPs-HRP-GCE sensor was fabricated by dropping 5 µL of the HRP solution onto the surface of the MWCNT-TiO_2_NPs-GCE. The modified electrode was dried and then stored in the refrigerator at 4 °C [23].

### 2.5. Electrochemical Measurements with the MWCNT-TiO_2_NPs-HRP-GCE

A total of 10 mL of the 0.1 M of PBS with pH 7.0 was added into the electrochemical cell in which the working electrode was immersed prior to the electrochemical measurements. The cell was purged with nitrogen gas for 5 min to remove the content of dissolved oxygen. To optimize the background current readings, several cyclic sweeps were carried out. The INZ sample was added into the electrochemical cell and stirred at 1000 rpm with a deposition time of 120 s, and equilibrating time of 5 s, and the sweeping was done at an optimum scan rate of 0.09 V·s^−1^ in differential pulse voltammetric (DPV) mode.

## 3. Results and Discussion

### 3.1. Characterization of the MWCNT-TiO_2_NPs-HRP-GCE

**Figure 1:**
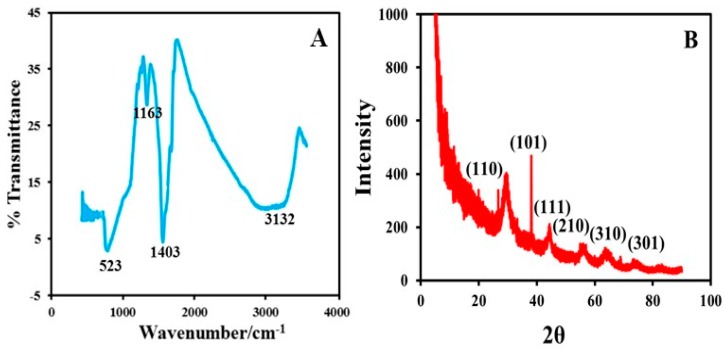
(**A**) Fourier transform infrared spectroscopy (FT-IR) characterization of titanium oxide nanoparticles (TiO_2_NPs). (**B**) X-ray diffraction (XRD) image of TiO_2_NPs.

The MWCNT-TiO_2_NPs-HRP-GCE was characterized using FT-IR, X-ray diffraction (XRD), transmission electron microscopy (TEM), and thermogravimetry (TGA). FT-IR was performed to assess characteristic stretching variations for synthesized TiO_2_NPs. Figure 1A reveals the presence of a sharp peak at 523 cm^−1^ indicating the presence of Ti-O stretching vibrations. A well-defined sharp peak appeared at 1403 cm^−1^, indicating the Ti-O-Ti stretching vibrations. The peak at 1163 cm^−1^ is responsible for C-O stretching [24]. In addition, peaks at 3132 cm^−1^ were attributed to the presence of O-H stretching. Figure 1B shows the XRD data, with Miller indices (101,110, and 111) attributed to the body-centred cubic, crystalline nature of the TiO_2_NPs. The broad diffraction peaks are an indication of the nano-size of crystalline compound. The experimental XRD pattern agrees with the 2θ peak at 25.4° and confirms the TiO_2_ anatase structure [25]. The strong diffractions at 25° and 48° were also attributed to TiO_2_NPs in the anatase phase [26]. The above evidence confirmed the synthesized TiO_2_NPs.

**Figure 2:**
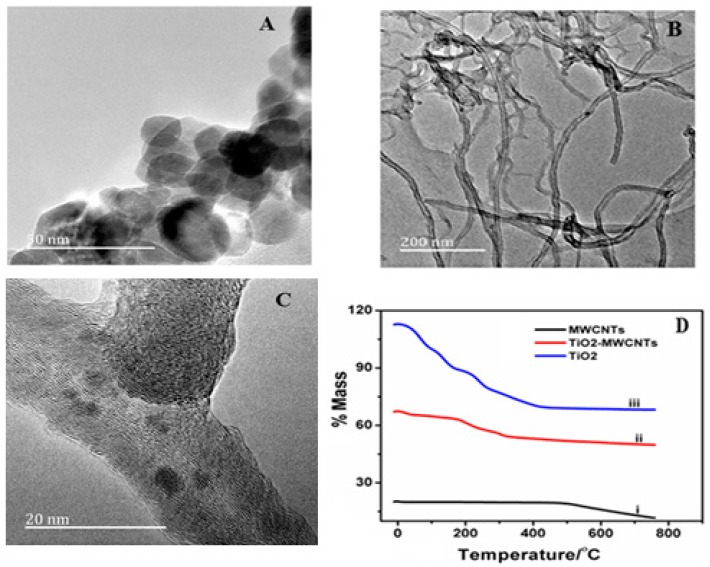
(**A**) Transmission electron microscopy (TEM) image of TiO_2_NPs (**B**) Pure multiwalled carbon nanotubes (MWCNTs) (**C**) MWCNTs-TiO_2_NPs (**D**) Thermogravimetry (TGA) curves for: (1) MWCNTs; (2) MWCNTs-TiO_2_NPs; and (3) TiO_2_NPs.

Figure 2A is the TEM image of the TiO_2_NPs, which clearly indicates the spherical shape geometry of TiO_2_NPs with an average diameter of 25 nm. Figure 2B shows TEM of pure MWCNTs as having tubular network-like structure. Figure 2C clearly shows the adherence of TiO_2_NPs on the surface of the MWCNTs. Figure 2D shows the thermogravimetric curves of pure MWCNTs, TiO_2_NPs and MWCNTs-TiO_2_NPs. The TGA curve of MWCNTs (black line in Figure 2D) exhibited definite mass loss at 570 °C, which may possibly be due to the carbon oxidation. The TGA curve of TiO_2_NPs showed nearly flat characteristics with minor mass loss observed before 100 °C, and this may be due to loss of water, whereas at 400 °C this may signify loss of nitrogen content. The TGA curve of MWCNTs-TiO_2_NPs also showed slight mass loss in two stages around at 100 °C and 250 °C, respectively; this may be due to the evaporation of solvent. Beyond that temperature, the composite was highly stable due to the dispersion of TiO_2_NPs on the surface of MWCNTs.

### 3.2. Method Optimization

**Figure 3:**
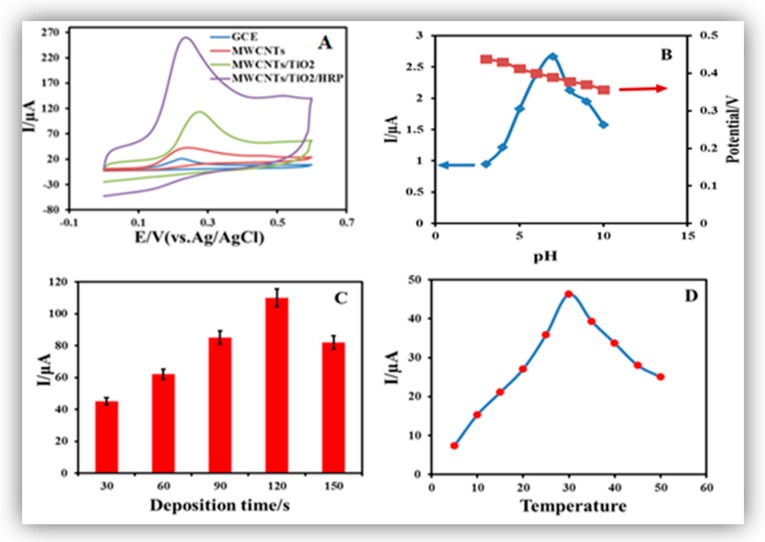
(**A**) Cyclic voltammograms of 0.1 mM isoniazid (INZ) with: (1) a bare glassy carbon electrode (GCE); (2) MWCNT-GCE; (3) MWCNT-TiO_2_NPs-GCE; and (4) MWCNT-TiO_2_NPs-horseradish peroxide (HRP)-GCE. (**B**) Peak potential and peak currents response with pH at 3.0, 4.0, 5.0, 6.0, 7.0, 8.0, 9.0 and 10.0. respectively (**C**) Peak current responses vs. different deposition times of 30, 60, 90, 120 and 150 s. (**D**) Effect of temperature on the current response of the MWCNT-TiO_2_NPs-HRP-GCE.

The effect of pH played an important role in the electrochemical signal amplification of INZ. The effect of pH with peak current was studied in the range of 3.0–10.0. As the pH increases, peak currents gradually increase until pH 7, and then decrease beyond pH 7. The changes in the anodic peak currents and peak potentials responses were monitored by varying the pH (Figure 3B). According to the obtained results from Figure 3B, the anodic peak current of INZ reaches a maximum at pH 7, and decreases beyond 7. Due to this observation, a pH of 7 was selected as optimum for the entire study. In addition, the effect of deposition time was also monitored from 30 to 150 s (Figure 3C). Based on the obtained results, deposition time with 120 s was selected as optimum deposition time for the present study. Further, the most effective parameter, notably, scan rate, was examined in the range of 0.1 to 1.0 V·s^−1^. The obtained results showed that 0.9 V·s^−1^ was the optimum potential scan rate. The effect of incubation temperature was also tested with the activity of HRP immobilized on the surface of the MWCNT-TiO_2_NPs-GCE. The anodic peak current response was observed with different incubated temperatures ranging from 5 to 50 °C (Figure 3D). The fabricated MWCNT-TiO_2_NPs-HRP-GCE showed maximum peak currents at 30 °C. Therefore, 30 °C was selected as the optimum temperature for the successful experiment. At 5–30 °C the increase in current responses of the modified sensor was due to the increase in the activation energy of the reaction. After 30 °C, the current responses were decreased due to enzyme denaturation. The enzyme reaction rate increases as the incubation temperature increases up to an optimum temperature, and after that the enzyme activity declines rapidly. Moreover, the immobilized enzyme shows no changes in the optimum temperature when compared with free enzyme. The enzyme incubation time is also an important parameter in the method optimization, which was monitored from 5 to 25 min at room temperature, and the corresponding current responses were measured. Figure S1 showed the current responses gradually increased with sustained enzyme incubation time. The enzyme-fabricated electrode showed good peak current response at 15 min. Based on the current response and adsorption equilibrium of the enzyme, 15 min was selected as the optimum incubation time for the entire study. After 15 min the current response gradually decreased with prolonged incubation time.

### 3.3. Electrochemical Behavior of INZ on the MWCNT-TiO_2_NPs-HRP-GCE

**Scheme 1:**
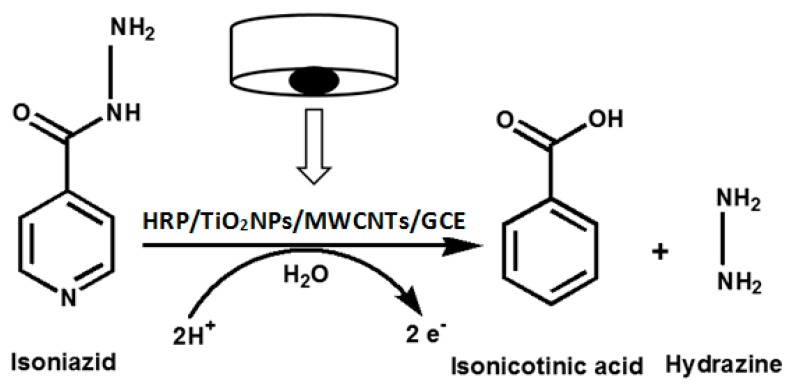
Electrochemical oxidation mechanism of INZ with the MWCNT-TiO_2_NPs-HRP-GCE.

The MWCNT-TiO_2_NPs-HRP-GCE was electrochemically analysed by CV and DPV techniques. The modified electrode surface area calculation was justified by using the Randles–Sevick [27] Equation (1).

(1) $$i_{pa}=2.69\times 10^{5}AC_{0}n^{3/2}D_{R}{}^{1/2}v^{1/2}$$

where $i_{pa}$ is the anodic peak current, *A* is the surface area of the electrode, *C*_0_ is the concentration of INZ, *n* is the number of electrons transferred, *D_R_* is the diffusion coefficient, and *V* is the scan rate. Based on Equation (1), the MWCNT-TiO_2_NPs-HRP-GCE and GCE surface areas were calculated and found to be 19.32 and 3.14 mm^2^, respectively. This indicated that the MWCNT-TiO_2_NPs-HRP-GCE sensor was exhibiting approximately a six times larger surface area than the GCE. This implies that the fabricated sensor provides a high surface area for INZ to undergo electrochemical oxidation [28] (Scheme 1).

**Figure 4:**
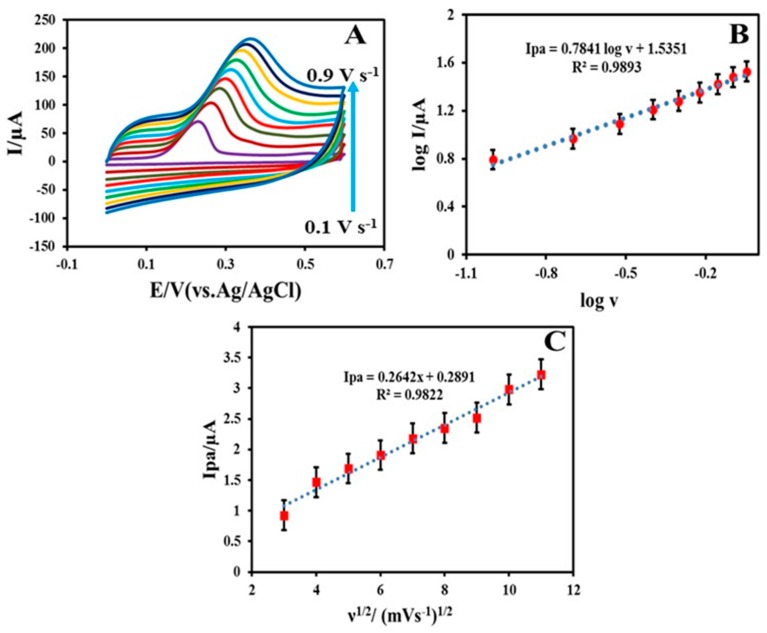
(**A**) Cyclic voltammograms of 0.1 mM of INZ at scan rates of 0.1, 0.2, 0.3, 0.4, 0.5, 0.6, 0.7, 0.8 and 0.9 V·s^−1^. (**B**) Graph of (log anodic peak current) versus log (scan rate) for 0.1 mM INZ in 0.1 M phosphate buffer solution (PBS; pH 7.0) with different scan rates for the MWCNT-TiO_2_NPs-HRP-GCE. (**C**) Linear plot of (anodic peak current) against the square root of the scan rate (*n* = 5, average standard deviation).

The electrochemical sensing ability of the MWCNT-TiO_2_NPs-HRP-GCE was compared with the bare GCE, MWCNTs and MWCNTs-TiO_2_NPs (Figure 3A). The potentials scale was monitored in the range of −0.1 to +0.7 V for the appearance of anodic peak. The GCE exhibited a much lower current of 25 µA, while the MWCNT-GCE and MWCNT-TiO_2_NPs-GCE showed moderate current responses of 56 µA and 110 µA, respectively (Figure S2). The final MWCNT-TiO_2_NPs-HRP-GCE showed much greater peak current responses with 200 µA (Figure 4A). Figure 4B shows the linear relationship between log $i_{pa}$ and log *v*, and it is suggested the diffusion of INZ at the surface of the MWCNT-TiO_2_NPs-HRP-GCE can be expressed by the following Equation (2).

(2) $$\log\;i_{pa}=0.7841\;\log\;v+1.5351$$

Figure 4C shows that the oxidation of INZ at the MWCNTs-TiO_2_NPs-HRP-GCE is similar to the diffusion-controlled process owing to the linear relationship between anodic peak current and the square root of the scan rate. The fitted regression line can be expressed as *i_pa_* = 0.2642 *υ*^1/2^ + 0.2891; R^2^ = 0.9822. This clearly indicates the outstanding performance of the MWCNT-TiO_2_NPs-HRP-GCE compared to the other three electrodes.

### 3.4. Quantitative Analysis of INZ

**Figure 5:**
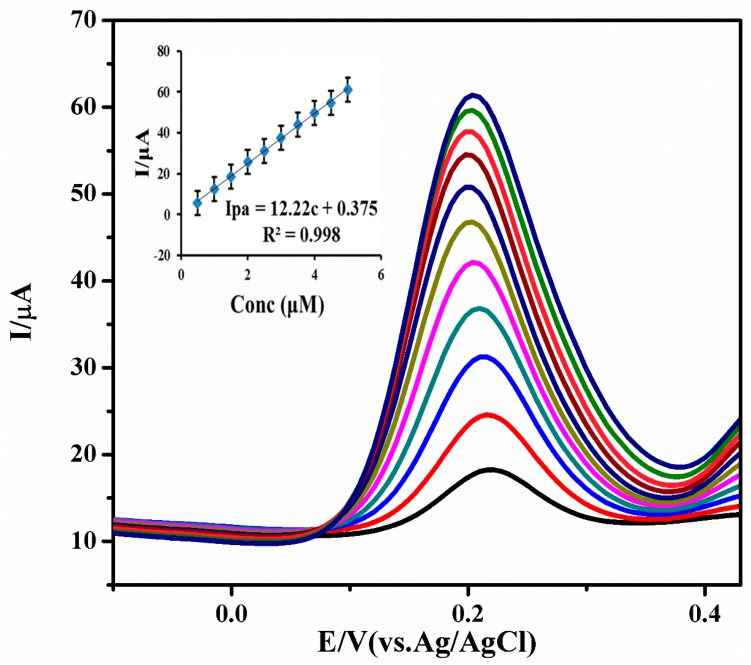
Differential pulse voltammetric (DPV) response of 0.5 to 5 μM INZ with the MWCNT-TiO_2_NPs-HRP-GCE at pH 7.0, scan rate: 0.09 V·s^−1^, deposition time: 120 s, pulse amplitude: 0.050 V and pulse time: 0.040 s. (Inset calibration plot) (*n* = 5, average SD).

DPV was carried out with a 0.5 to 5 μM of INZ, via the standard addition method. The MWCNT-TiO_2_NPs-HRP-GCE gave a well-defined anodic peak at 0.2 V (vs. Ag/AgCl) with the optimized working parameters (scan rate of 0.09 V·s^−1^, deposition time 120 s, pulse amplitude 0.050 V, voltage step 0.00935 V, voltage step time 0.1 s and pulse time 0.040 s). It was shown that as the concentration of INZ was increased, the anodic peak currents were gradually increased (Figure 5).

A calibration graph was plotted for the concentration of INZ against anodic peak currents. The obtained regression equation (*i_pa_* =12.22 *c* + 0.375) showed a good correlation coefficient of R^2^ = 0.998 for DPV. The limit of detection (LOD) and limit of quantification (LOQ) were calculated based on signal to noise ratios by using the Equations (3) and (4). In terms of the instrument signal, we are interested in determining the smallest signal that is distinguishable from the background (baseline) noise.

LOD = (3 × SD/Slope)(3)

LOQ = (10 × SD/Slope)(4)

where SD is the standard deviation of the peak currents for three different runs and slope of the calibration curve. The limit of detection and limit of quantification for INZ is 0.0335 μM and 0.1118 μM, respectively. The comparison of current fabricated sensor method with previous methods (27–37) used in the determination of INZ is listed in Table 1. This indicates our method showed lower LOD and LOQ values for the determination of INZ compared to previous reports.

### 3.5. Repeatability and Stability

The repeatability efficiency of the proposed sensor was examined with 0.1 mM INZ solution DPV under the optimized parameters. After each determination the used fabricated electrode underwent five to six sweeps in 0.1 M PBS (pH 7.0), to remove any adsorbents and yield a reproducible electrode surface. The peak current response of INZ was determined with five electrodes under the same conditions. From the five parallel determinations, the Relative Standard Deviation (RSD) of INZ determination was found to be 4.12%. Based on the results, the proposed sensor showed good repeatability. The long-term stability of the sensor was tested after 80 days. When not used, the sensor was stored at 4 °C. The stability studies were carried out with the MWCNT-TiO2NPs-HRP-GCE for the detection of INZ. This study was carried out in two batches. In the first batch, the stability of the modified electrode was observed from day 1 to day 40, in terms of electrochemical signalling. It was observed that for 40 days, the average electrochemical signal was found to be 91%. In the second batch, the stability study was carried out for an additional 40 days with the same materials and electrode. Interestingly, it was now observed that the electrochemical signal was found to be 87%. This implies only 4.39% of the electrochemical signal decreased. These results indicate that the fabricated electrode showed good repeatability and long-term stability.

### 3.6. Interference Studies

To evaluate the selectivity ability of developed sensor, interference studies were also performed for INZ with coexisting compounds like uric acid, glucose, and ascorbic acid. In this analysis, the peak current of INZ was recorded (Ip_1_). The excess amount of the potential interferent species was added to the mixture and DPV was recorded (Ip_2_). The tolerance limit was defined as the maximum concentration of the interfering components that caused an error less then ±5% (Ip_1_/Ip_2_ = 95–105%). In addition, the influence of some common ions such as Fe^3+^, Al^3+^, Cl^−^, Na^+^, and K^+^ were studied and the results indicated that these ions have no significant influence on the determination of INZ (Table 2).

### 3.7. Real Sample Analysis

Commercially-available INZ tablets (100 mg) were purchased from the local pharmacy. The samples (tablets) were used to evaluate the realistic performance of the MWCNT-TiO_2_NPs-HRP-GCE. Approximately ten tablets were taken into a mortar and crushed to fine powder with pestle. A 0.1 mM INZ tablet sample solution was prepared by dissolving the appropriate amount of powder sample in a 10 mL volumetric flask and adding 10 mL of PBS. Finally, real sample analysis was carried out with 0.1 mM INZ by the DPV technique via the standard addition method. The results with respect to the analytical performances of the MWCNT-TiO_2_NPs-HRP-GCE obtained were then tabulated (Table 3). The two pharmaceutical samples showed good RSD values (1.69 and 1.98) with an excellent percentage of recovery (99.2% and 98.9%). This indicates that the developed sensor exhibited good recovery capability towards INZ in various pharmaceutical samples.

## 4. Conclusions

A facile MWCNT-TiO_2_NPs-HRP-GCE biosensor was successfully developed for the determination of INZ in different pharmaceuticals samples. The synthesized nanoparticles and developed sensor were characterized with TGA, TEM, XRD, and FT-IR. The electrochemical sensing abilities of INZ with different electrodes, viz., bare GCE, MWCNT-GCE, MWCNT-TiO_2_NPs-GCE and MWCNT-TiO_2_NPs-HRP-GCE were successfully compared using cyclic voltammetry studies. The developed sensor showed fast electron transfer capability, good electronic conductivity, and large electroactive surface area. The CV results obtained reveal the outstanding electrochemical sensing performance of the enzyme-induced sensor with an increase (approximately 8-fold) of anodic peak currents. In the DPV technique, the calibration plot gave a good correlation coefficient of R^2^ = 0.998 with LOD and LOQ values of 0.0335 μM and 0.1118 μM, respectively. This clearly indicates the developed sensor showed a high capability for detecting the INZ in μM concentration without any interferences. In addition, the fabricated sensor exhibited good repeatability and long-term stability, with negligible current variations. Moreover, the real sample analysis gave satisfactory results with good percentages of recoveries. The devolved sensor may have scope for use in the pharmaceutical industries in the near future.

## Figures and Tables

**Table 1:** Comparison of some characteristics of the previously reported modified electrodes with the MWCNT-TiO_2_NPs-HRP-GCE.

Electrode	Technique	Detection Limits/(μM)	Linear Range/(μM)	Buffer and pH	References
Nf/Fe/GCE ^a^	CV	13.00	50–20,000	Na_2_SO_4_ (9)	[29]
LDH/GCE ^b^	DPV	4.00	4.9–770	BR (9)	[30]
4-pyridyl hydroquinone SAM/platinum electrode ^c^	CV	20.0	-	PBS (7.2)	[31]
poly-L-histidine/SPE ^d^	DPV	0.50	-	PBS (7)	[32]
Gold electrode ^e^	DPV	0.09	-	NaOH (13.6)	[33]
PdNP/CPE ^f^	CV	0.47	-	PBS (7)	[27]
Hanging mercury drop electrode ^g^	SWADCS	1.18	-	BR (5.5)	[34]
GO/GCE ^h^	LSV	0.17	2–70	PBS (7)	[35]
F-MWCNT/GCE ^i^	CV	0.27	1–70	AB (4)	[36]
Rh/GCE ^j^	CV	13.00	70–130	PBS (7)	[37]
Bentonite clay/GCE ^k^	LSV	0.80	-	Na_2_SO_4_ (13.5)	[38]
MWCNT-TiO_2_NPs-HRP-GCE	DPV	0.03	0.5–5	PBS (7)	Present work

^a^ Nf/Fe/GCE—Nafion on iron-coated GCE; ^b^ LDH/GCE—layered double hydroxide-coated GCE; ^c^ 4-pyridyl hydroquinone SAM/platinum electrode-4-pyridyl hydroquinone self-assembled monolayer-coated platinum electrode; ^d^ poly-L-histidine/Screen printed electrode (SPE)-poly-L-histidine coated on a SPE ; ^f^ PdNP/CPE—palladium nanoparticle-coated CPE; ^h^ GO/GCE—graphene oxide-coated GCE; ^i^ F-MWCNT/GCE—functionalized multi-wall carbon nanotube-coated GCE; ^j^ Rh/GCE—rhodium nanoparticle-coated GCE; ^k^ bentonite clay/GCE—bentonite clay-modified GCE; SWADCS—square-wave adsorptive anodic stripping voltammetry; LSV—linear sweep voltammetry; CPE—carbon paste electrode; SPE—screen-printed electrode; CV—cyclic voltammetry; PBS—phosphate buffer solution.

**Table 2:** The influences of some anions, cations and important biological substances on the peak current of 0.1 mM INZ with the MWCNT-TiO_2_NPs-HRP-GCE.

Interferents	Concentration/(µM)	Signal Change (%)
Ascorbic acid	250	4.03
Uric acid	250	1.24
Glucose	500	0.34
Fe^3+^	500	0.81
Al^3+^	500	1.27
Cl^−^	500	0.67
Na^+^	500	2.01
K^+^	500	1.35

**Table 3:** Determination of INZ in various pharmaceutical samples using the developed sensor (*n* = 5).

Declared Amount Tablet (mg)	Found (mg)	Recovery (%)	Relative Standard Deviation (RSD) (%)	Added (mg)	Found (mg)	Recovery (%)	RSD (%)
Sample 1 (100 mg)	99.2	99.2	1.69	20	19.3	96.5	0.32
Sample 2 (100 mg)	98.9	98.9	1.98.	10	9.5	95.0	0.79

*n*: average determinations.

## Supplementary Materials

The following are available online at http://www.mdpi.com/2079-6374/7/2/21, Figure S1: The effect of the enzyme incubation time on the current responses of modified electrode. Figure S2: Cyclic Voltammograms of INZ with (A) Bare GCE, (B) MWCNTs-GCE, (C) MWCNTs-TiO_2_NPs-GCE at various scan rates ranges 0.1, 0.2, 0.3, 0.4, 0.5, 0.6, 0.7, 0.8 and 0.9 V·s^−1^
