# Supplementary material for: An Enzyme-Induced Novel Biosensor for the Sensitive Electrochemical Determination of Isoniazid
Source: Biosensors (Basel). 2017 Jun 5;7(2):21. doi: 10.3390/bios7020021 (PMC5487961; doi:10.3390/bios7020021)
Supplement: Supplementary file 1 [file biosensors-07-00021-s001.pdf]

Supplementary Materials: An Enzyme-Induced Novel Biosensor for the Sensitive Electrochemical Determination of Isoniazid

Rajasekhar Chokkareddy, Natesh Kumar Bhajanthri, Gan G. Redhi

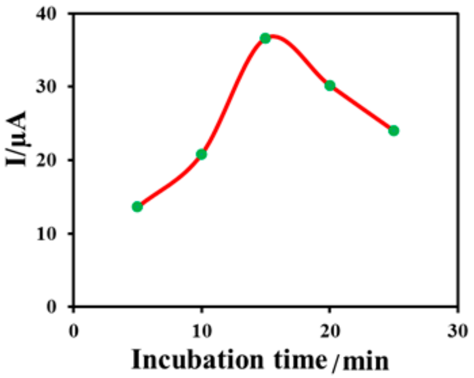

Figure S1. The effect of the enzyme incubation time on the current responses of the modified electrode.

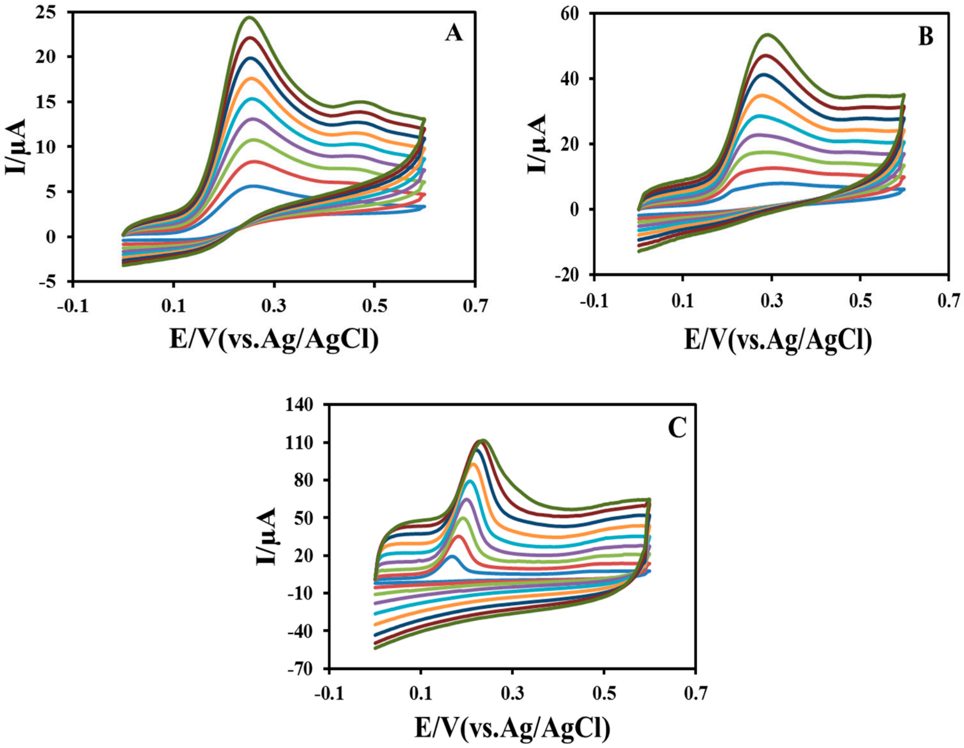

Figure S2. Cyclic Voltammograms of INZ with (**A**) Bare GCE, (**B**) MWCNT-GCE, (**C**) MWCNT-TiO_2NP-GCE at various scan rates: 0.1, 0.2, 0.3, 0.4, 0.5, 0.6, 0.7, 0.8 and 0.9 $V \cdot s^{-1}$.
